# Odontogenic tumors: clinical and pathology study of 238 cases

**DOI:** 10.1016/S1808-8694(15)31375-6

**Published:** 2015-10-17

**Authors:** Rafael Linard Avelar, Antonio Azoubel Antunes, Thiago de Santana Santos, Emanuel Sávio de Souza Andrade, Edwaldo Dourado

**Affiliations:** 1 Dental surgeon, resident in buccomaxillofacial surgery and traumatology, Hospital Universitário Oswaldo Cruz; 2 Dental surgeon, student in the specialization course on buccomaxillofacial surgery and traumatology, Universidade de Pernambuco - FOP/UPE; 3 Dental surgeon, resident in buccomaxillofacial surgery and traumatology, Hospital Universitário Oswaldo Cruz - HUOC/UPE; 4 Doctor, adjunct professor of buccal pathology, Faculdade de Odontologia de Pernambuco - Universidade de Pernambuco - FOP/UPE; 5 Doctor, adjunct professor of buccomaxillofacial surgery and traumatology, Faculdade de Odontologia de Pernambuco - Universidade de Pernambuco - FOP/UPE. Discipline of buccal pathology, Faculdade de Odontologia da Universidade de Pernambuco - FOP/UPE

**Keywords:** classification, epidemiology, odontogenic tumors

## Abstract

Odontogenic tumors are neoplasms that develops exclusively in the gnathic bones; they originate from odontogenic tissues, by epithelial or mesenchymal proliferation, or both.

**Aim:**

To evaluate the incidence of odontogenic tumors in a specific institution, and to compare these findings with other studies in the literature.

**Study format:**

A cross-sectional cohort retrospective study.

**Material and method:**

The sample was obtained from the files of patients with odontogenic tumors diagnosed between January 1992 and March 2007 (15 years). Cases in which the diagnosis could be adapted to the new World Health Organization (WHO) of 2005 were included. Data such as gender, age, anatomical site, histological type and symptomatology were analyzed.

**Results:**

Odontogenic tumors were 4.76% of all biopsied lesions within the studied period. The mean age was 30.7 years; 57% of the patients were male. The keratocystic odontogenic tumor was the most prevalent histological type (30%), followed by the ameloblastoma (23,7%). The rate of asymptomatic cases was 75.7%.

**Conclusion:**

Odontogenic tumors occurred more frequently in females, in the second and third decades of life, and more commonly in the mandible; most cases were asymptomatic.

## INTRODUCTION

Odontogenic tumors are a heterogeneous group of lesions with variable clinical and pathohistological features. The biological behavior of these tumors includes hamartomatous proliferation, non-aggressive benign tumors, and aggressive and malignant tumors.^1^ There has been considerable interest in odontogenic tumors by oral pathologists, who have studied and catalogued these tumors for decades. These tumors are 2.5% of all biopsied lesions in dental offices.^2^, ^3^

Although many retrospective studies have been conducted in Africa,^4^, ^5^, ^6^ Asia,^7^ Europe,^8^, ^9^ and North America,^10^ unanswered question still remain about the relative frequency and the incidence of certain odontogenic tumors.^11^

The geographical distribution of these lesions is variable.^2^ Many studies in different part of the world have shown differences in the relative prevalence of these tumors.^12^ Few reports have been published in English about the frequency of odontogenic tumors in Latin America, particularly in Brazil.^13^ The frequency was 1.29%^14^ in a Chilean study of 362 cases.

Various attempts at classification of these tumors have been published to define diagnostic criteria, given the diversity of lesions that may arise from odontogenic tissues.1

The first classification of these tumors was published in 1971, based on a 5-year joint effort coordinated by the World Health Organization (WHO).12 An updated edition of this classification was published in 1992.^15^ A new classification was proposed in 2005, which included the odontogenic keratocyst as a benign odontogenic tumor.^16^

The purpose of this study was to investigate the epidemiological behavior of this heterogeneous group of tumors across a 15-year period (1992–2007) and to compare these data with those in the literature.

## MATERIAL AND METHODS

A retrospective study was made of cases of odontogenic tumors recorded at our institution between January 1992 and March 2007. The variables gender, age, anatomical site, histological type and symptoms were analyzed in 238 histopathology reports.

In reports with recurring tumors, the histological appearance of the first and recurring tumors was compared and considered as a single case.

The diagnoses were reassessed and adapted to the 2005 WHO classification.^16^

After the sample was obtained, a database was generated using the SPSS (v. 13.0) statistics software; the chi-square test was applied to check the statistical significance of the findings. A p value below 0.05 was considered statistically significant.

This study was duly recorded by the Research Ethics Committee of our institution (protocol number 135717/07).

## RESULTS

Patients were distributed according to the data as follows:

## DISCUSSION

Odontogenic tumors are infrequent in gnathic bones, and should be included in the differential diagnosis of gnathic bone lesions.^2^ Few published studies have been done on large series in any country or region to assess age, gender and site of odontogenic tumors, based on the 1992 classification of the World Health Organization.^3^, ^6^, ^7^, ^14^, ^17^

As of 2005, a new classification included the odontogenic keratocyst as one of the odontogenic tumors, renaming it as a keratocystic odontogenic tumor. This was the most prevalent lesion in this study (30%) (Table 2); given its recent reclassification, it was not possible to compare its prevalence with that of other odontogenic tumors or with findings published elsewhere. The incidence was slightly higher in females (Table 1); this finding has not been demonstrated in other studies, such as that by Mosqueda-Taylor et al.^18^ There were two age-related peaks in both genders; the highest peak was in the second and third decades of life, and a lower peak occurred in the seventh decade of life (Table 2). These findings are similar to those of Ahlfors et al.^19^

**Table 2:** Age of patients with odontogenic tumors.

Histological types			Age Groups								Total	
	Age ranges	Mean ages	00–10	11–20	21–30	31–40	41–50	51–60	61–70	70	n	%
Ameloblastoma	10–99	34	1	14	16	8	5	9	3	1	57	23,7
Cementoblastoma	40–57	44	-	-	-	2	1	1	-	-	4	1,7
Keratocystic Odontogenic Tumor	10–84	31	2	14	23	15	9	3	2	1	69	30,0
Calcifying Epithelial Odontogenic Cyst	10–70	32	1	4	5	-	1	3	1	-	15	6,3
Ameloblastic Fibroma	38–45	42	-	-	-	1	3	-	-	-	4	1,7
Ameloblastic Fibrodontoma	11	11	-	1	-	-	-	-	-	-	1	0,4
Mixoma	7–45	29	1	4	3	4	3	-	-	-	15	6,3
Odontoma	4–84	27	8	17	10	9	4	2	2	2	54	22,1
Adenomatoid Odontogenic Tumor	10–45	20	1	7	2	2	1	-	-	-	13	5,4
Calcifying Epithelial Odontogenic Tumor	11–46	26	-	3	-	1	1	-	-	-	5	2,0
Squamous Odontogenic Tumor	16	16	-	1	-	-	-	-	-	-	1	0,4

p>0,05 - chi-square test

**Table 1:** Distribution of patients according to gender.

Histological types	Gender			
	Male		Female	
	N	%	n	%
Ameloblastoma	30	52,6	27	47,4
Cementoblastoma	1	25	3	75
Keratocystic Odontogenic Tumor	30	43,4	39	56,6
Calcifying Epithelial Odontogenic Cyst	7	46,6	8	53,4
Ameloblastic Fibroma	3	75	1	25
Ameloblastic Fibrodontoma	-	-	1	100
Mixoma	8	53,4	7	46,6
Odontoma	17	31,4	37	68,6
Adenomatoid Odontogenic Tumor	3	23	10	77
Calcifying Epithelial Odontogenic Tumor	3	60	2	40
Squamous Odontogenic Tumor	-	-	1	100

TOTAL	102		136	

p>0,05 - chi-square test

The ameloblastoma was the second most common tumor in this study (23.7%) (Table 2); its frequency was lower compared to published reports by Lu et al.^7^ (59%), Oduyoka6 (58%) and Adebayo et al.^5^ (48%). The ameloblastoma is considered as the most common histological type in Africa, followed by the odontogenic mixoma.^6^, ^15^ In countries like Chile^18^ and Canadá,^10^ they are respectively 20 and 18% of such tumors; the most frequent tumor in these countries is the odontoma (Chile - 45 and Canada - 46%).^10^, ^19^ In our study, the frequency of ameloblastoma was higher in males (52.6%) (Table 1), as also seen in studies conducted in Nigeria5 and Turkey;^8^ most of these patients had no tumor-related symptoms (79%; p=0.023) (Table 4). Findings in African studies have shown that the prevalence of this tumor is higher in the second to fourth decades of life,^5^, ^17^ and that they are located preferably in the mandible,^7^, ^17^ both of which were confirmed in our study (Tables 2 and 3).

**Table 4:** Distribution of patients according to symptoms.

Histological types	Symptoms			
	Symptomatic		Asymptomatic	
	N	%	n	%
Ameloblastoma	12	21	45	79
Cementoblastoma	2	50	2	50
Keratocystic Odontogenic Tumor	20	28,9	49	71,1
Calcifying Epithelial Odontogenic Cyst	3	20	12	80
Ameloblastic Fibroma	1	25	3	75
Ameloblastic Fibrodontoma	1	100	-	-
Mixoma	4	26,6	11	73,4
Odontoma	10	18,5	44	81,5
Adenomatoid Odontogenic Tumor	3	23	10	77
Calcifying Epithelial Odontogenic Tumor	1	20	4	80
Squamous Odontogenic Tumor	1	100	-	-

TOTAL	58		180	

p=0,023 - chi-square test

**Table 3:** Anatomical sites of odontogenic tumors.

Histological types	Anatomical site			
	Mandible		Maxilla	
	N	%	n	%
Ameloblastoma	48	84,2	09	15,8
Cementoblastoma	04	100	-	-
Keratocystic Odontogenic Tumor	52	75,3	17	14,7
Calcifying Epithelial Odontogenic Cyst	10	66,6	05	33,3
Ameloblastic Fibroma	04	100	-	-
Ameloblastic Fibrodontoma	01	100	-	-
Mixoma	07	46,6	08	53,4
Odontoma	23	42,5	31	57,5
Adenomatoid Odontogenic Tumor	06	46,1	07	53,9
Calcifying Epithelial Odontogenic Tumor	04	80	01	20
Squamous Odontogenic Tumor	01	100	-	-

TOTAL	160		78	

p>0,05 - chi-square test

Odontomas are 4% to 67% of odontogenic tumors.^5^, ^6^, ^10^ This was the most common of these tumors in the Americas, as reported by Ochsenius et al.,^14^ Mosqueda-Taylor et al.,^13^ and Daley et al.;^10^ odontomas are least frequent in Africans^6^ and Chinese.^7^ There were 54 cases of odontomas in our 15-year study (22.1%) (Table 2). The lower incidence of odontomas in Africans^4^ is due probably to the lack of symptoms of many of these lesions^6^ or by genetic factors. This neoplasm was diagnosed mostly in patients aged below 30 years; published papers have reported that new odontomas are discovered up to the third decade of life.^5^, ^6^ Odontomas were more prevalent in the maxilla (57.5%) (Table 3) and in females (68.6%) (Table 1); this is similar to the findings of Santos et al.^13^

The incidence of the odontogenic mixoma was 6.3% of all odontogenic tumors in our study (Table 2), which is about two times less than that reported by Mosqueda-Taylor et al.,^3^ but comparable to North-American studies.^10^ Our study revealed a slightly higher prevalence of this tumor in males (Table 1); Ladeinde et al.^12^ and Odukoia et al.^6^ have reported a higher frequency in females. This study showed that the mean age of these patients was 29 years, higher than the age reported in two previous studies in Nigeria^5^ and Mexico,^11^ in which the mean age was 19 years. A slight preference for the maxilla (53.4%) (Table 3) has been reported in a number of studies, such as those by Mosqueda-Taylor et al.;^3^ Lu et al.,^7^ on the other hand, have reported differently.

The incidence of the adenomatoid odontogenic tumor was 5.4% of all odontogenic tumors in our study (Table 2). Some papers have reported that this tumor is more frequently found in the maxilla^4^, ^13^, ^17^ and in female patients.^17^ (Table 1); these findings were confirmed in our study, in which 53.9% of these lesions were found in the maxilla (Table 3) and 77% of these patients were female (Table 1), mostly in the second decade of life (7 of 13 cases) (Table 2).

The calcifying epithelial odontogenic cyst was seen in 15 cases (6.3%) (Table 2); there was a slightly higher incidence in females (53.4%) (Table 1), and 66% of these cases were in the mandible (Table 3). These findings are similar to those in most studies^13^ except for Hiroyuki et al.’s1^7^ report. This neoplasm is usually found accidentally in routine exams.5 In our study, 80% of these cases were asymptomatic (p=0.023) (Table 4) and showed no age preference (Table 2).

The incidence of the ameloblastic fibroma and the cementoblastoma was 1.7% each, and the incidence of the calcifying epithelial odontogenic tumor was 2% of the odontogenic tumors in our study (Table 2). The cementoblastoma was more frequent in females (75%) (Table 1); the ameloblastic fibroma was more frequent in males (75%) (Table 1), as was the calcifying epithelial odontogenic tumor (60%) (Table 1). These lesions were more frequent in the mandible in our study (Table 3), contrary to some studies that have reported a higher frequency these tumors in the maxilla.^13^

Lesions such as the squamous odontogenic tumor and the ameloblastic fibrodontoma were found in a single case each; both were females and presented mandibular pain. Our study confirmed the low frequency of these neoplasms as shown in other studies,^3^, ^7^, ^12^, ^14^ which underlines their rarity.

It is essential to define the epidemiology of these tumors to improve our knowledge about their behavior, which allows us to optimize the diagnosis and therapy.

## CONCLUSION

There is a slight predominance of odontogenic tumors in women and during the first decades of life; most of them occur in the mandible, and most are asymptomatic. There were statistically significant differences among the variables histological type and symptoms.

In Brazil, specifically in this study, which was done in a region where population miscegenation is significant, there were some differences compared to published studies undertaken in other parts of the world.
